# Ectonucleotidase activity and immunosuppression in astrocyte-CD4 T cell bidirectional signaling

**DOI:** 10.18632/oncotarget.6914

**Published:** 2016-01-13

**Authors:** Fabia Filipello, Davide Pozzi, Michele Proietti, Andrea Romagnani, Sonia Mazzitelli, Michela Matteoli, Claudia Verderio, Fabio Grassi

**Affiliations:** ^1^ Laboratory of Pharmacology and Brain Pathology, Humanitas Clinical and Research Center, Rozzano, Italy; ^2^ Institute for Research in Biomedicine, Bellinzona, Switzerland; ^3^ Graduate School for Cellular and Biomedical Sciences, University of Bern, Bern, Switzerland; ^4^ CNR Institute of Neuroscience, Milano, Italy; ^5^ Department of Medical Biotechnology and Translational Medicine, University of Milan, Istituto Nazionale di Genetica Molecolare, Milan, Italy; ^6^ Center of Chronic Immunodeficiency, University Medical Center, Freiburg, Germany; ^7^ Hertie Institute for Clinical Brain Research, University of Tubingen, Department of Cellular Neurology, Tubingen, Germany

**Keywords:** astrocyte, ectonucleotidase, calcium, immunosuppression, T cell, Immunology and Microbiology Section, Immune response, Immunity

## Abstract

Astrocytes play a crucial role in neuroinflammation as part of the glia limitans, which regulates infiltration of the brain parenchyma by leukocytes. The signaling pathways and molecular events, which result from the interaction of activated T cells with astrocytes are poorly defined. Here we show that astrocytes promote the expression and enzymatic activity of CD39 and CD73 ectonucleotidases in recently activated CD4 cells by a contact dependent mechanism that is independent of T cell receptor interaction with class II major histocompatibility complex (MHC). Transforming growth factor-β (TGF-β) is robustly upregulated and sufficient to promote ectonucleotidases expression. T cell adhesion to astrocyte results in differentiation to an immunosuppressive phenotype defined by expression of the transcription factor Rorγt, which characterizes the CD4 T helper 17 subset. CD39 activity in T cells in turn inhibits spontaneous calcium oscillations in astrocytes that correlated with enhanced and reduced transcription of CCL2 chemokine and Sonic hedgehog (Shh), respectively. We hypothesize this TCR-independent interaction promote an immunosuppressive program in T cells to control possible brain injury by deregulated T cell activation during neuroinflammation. On the other hand, the increased secretion of CCL2 with concomitant reduction of Shh might promote leukocytes extravasation into the brain parenchyma.

## INTRODUCTION

The blood brain barrier (BBB) contributes to the immune privilege of the central nervous system (CNS) by excluding potentially harmful cells and macromolecules from the CNS parenchyma. Immunosurveillance of CNS is operated by effector T cells, which migrate across the BBB and are retained in cerebrospinal fluid (CSF)- drained perivascular and submeningeal spaces. Therein effector T cells remain separated from the CNS by glia limitans, a functional barrier composed of parenchymal basement membrane and astrocyte foot processes, located at interfaces between non-neural tissue and CNS parenchyma [1, 2]. Encountering of antigen presenting cells and effective *in situ* antigen recognition by the T cell, as observed in experimental autoimmune encephalomyelitis (EAE), result in upregulation of proinflammatory cytokines, proteases, chemokines, chemokine receptors as well as activation markers, breaching of glia limitans and neuroinflammation. In contrast, antigen ignorant T cells do not upregulate activation markers or pro-inflammatory cytokines and do not infiltrate the brain parenchyma [3].

The plasma membrane ecto-5′-nucleotidase CD73, an enzyme of the purine catabolic pathway that catalyzes the breakdown of AMP to adenosine, is induced in activated CD4 cells by TGF-β [4]. Extracellular adenosine generated by CD73 enzymatic activity contributes to immunosuppression by T regulatory (Treg) cells and might play a pivotal role in preventing autoimmune diseases [5, 6]. The rate-limiting step of the ectonucleotidase cascade for adenosine generation is represented by ectonucleoside triphosphate diphosphohydrolase 1 (E-NTPDase 1) CD39 that hydrolyzes ATP/UTP and ADP/UDP to the respective nucleoside (e.g., AMP). T cell receptor (TCR) stimulation induces CD39 enzymatic activity in the plasma membrane of mouse Treg cells [7], suggesting generation of adenosine through CD39 and CD73 is important for immunosuppression. On the other hand, adenosine generation by CD73 in the CNS is required for efficient entry of encephalitogenic lymphocytes into the brain and spinal cord during EAE [8].

Although serving as a barrier, which restricts the entry of inflammatory cells into CNS parenchyma [9-11], astrocytes have powerful pro-inflammatory potential. Also, dysfunction of astrocytes at the border of inflamed tissue leads to spread of neurotoxic inflammation into adjacent neural parenchyma. Thus, astrocytes are emerging as important regulators of neuroinflammatory events [2]. In this paper we show that antigen-independent adhesion of recently activated CD4 cells to astrocytes results in robust upregulation of plasma membrane CD39 and CD73 ectonucleotidases as well as T cell polarization to a Th17-like immunosuppressive phenotype. On the other hand, hydrolysis of extracellular ATP by CD39 expressed in T cells results in inhibition of ATP-dependent spontaneous calcium signaling and transcriptional regulation in astrocytes. We propose that this signaling pathway might constitute a regulatory mechanism for pro-inflammatory activation of antigen-specific T cells in the brain.

## RESULTS

### Expression of ectonucleotidases CD39 and CD73 in brain infiltrating CD4 cells in contact with astrocytes

**Figure 1:**
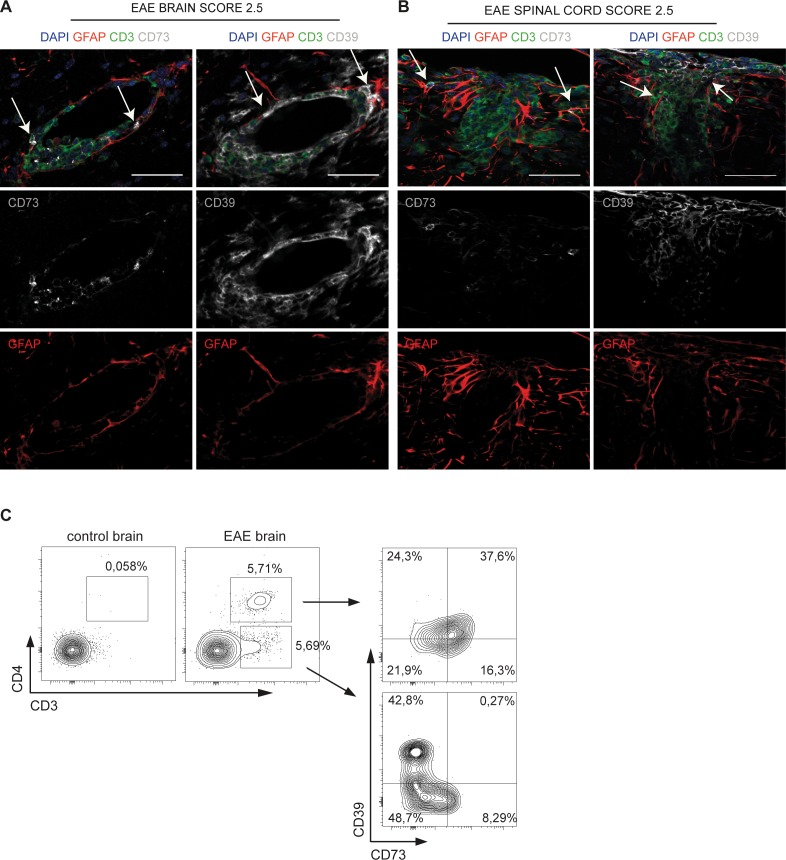
*In vivo* adhesion of ectonucleotidase positive T cells to astrocytes **A.**, **B.** Representative coronal sections of the brain (A) and spinal cord (B) of non-relapsing EAE at 20 days post-immunization. Immunofluorescence staining shows CD3^+^ (green), CD73^+^ or CD39^+^ (grey) cells and GFAP^+^ astrocytes (red). Arrows in A indicate CD73^+^ and CD39^+^ CD3^+^ cells contacting astrocytes at the outer perivascular area of blood vessels in stratum subependymal of lateral ventricles. Arrows in B show the same interactions in the spinal cord (caudal sections). These results are representative of 3 mice per organ. Scale bar, 50 μm. **C.** Analysis at FACS of CD39 and CD73 expression in T cells isolated *ex vivo* from control and EAE brains.

Autoantigen specific activation of CD4 cells is used to induce EAE in mice. This experimental model fairly reproduces neuroinflammation determined by pathogenic T cell activation in multiple sclerosis (MS). Confocal analysis of brain and spinal cord from EAE mice revealed infiltrating CD3^+^ T cells around blood vessels (Figure 1A) and in the spinal cord (Figure 1B) that were positive for both CD39 and CD73 ectoenzymes in the plasma membrane. Interestingly, CD39 or CD73 positive T cells were in direct contact with astrocytes as indicated by triple labeling for the astrocyte marker glial fibrillary acidic protein (GFAP) (arrows in Figure 1A, 1B). *Ex vivo* analysis of brain from mice with EAE in flow cytometry confirmed the presence of CD39^+^CD73^+^ double positive cells within the CD4^+^ subset and a population of CD39^+^ cells within the CD3^+^CD4^−^ compartment infiltrating the brain that were absent in healthy animals (Figure 1C).

### Upregulation of CD39 and CD73 in activated CD4 cells upon interaction with astrocytes

**Figure 2:**
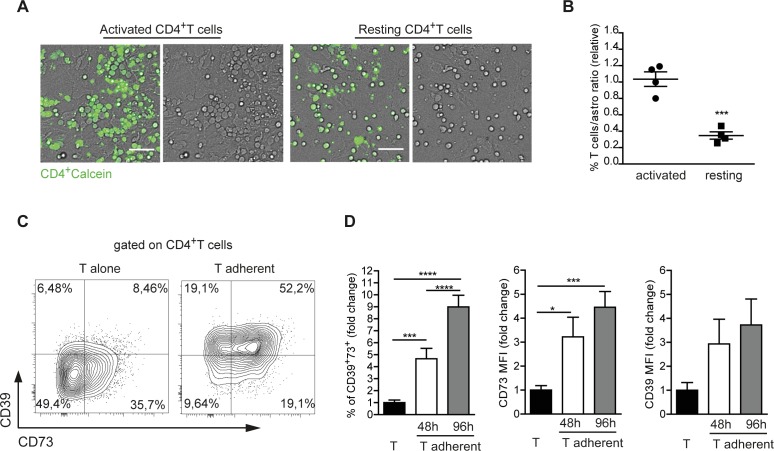
Astrocytes promote CD39 and CD73 expression in recently activated CD4 cells **A.** Co-cultures of CD4^+^ T cells loaded with calcein-AM (green) with purified astrocytes. Activated T cells massively adhered to astrocytes 48 h after plating, acquiring a flat shape (left), while resting (unstimulated) T cells remained suspended over the astrocyte monolayer (right). Scale bar 50 μm. **B.** Statistical analysis of resting *vs* activated T cells to astrocytes ratio after accutase treatment at 48h of co-culture. Student *T* test, ***p < 0.001. **C.** Representative dot plot of CD39 and CD73 expression in activated CD4^+^ T cells after 48 h of culture in isolation or adherent to astrocytes. **D.** Fold change in percentage of CD39^+^73^+^ T cells adherent to astrocytes, CD73 and CD39 MFIs after 48h (white) and 96h (grey) co-culture. Values were normalized to T cells alone (black). One-way ANOVA; Bonferroni's test for comparison among groups, *p < 0.05 ***p < 0.001, ****p < 0.0001; *N* > 6.

Since astrocytes are the first neural cells encountered by lymphocytes entering the brain, the above observation prompted us to investigate whether astrocytes may regulate surface expression of CD39 and CD73 in CD4 cells. Co-cultures of purified murine astrocytes with either resting or pre-activated CD4 T cells showed that a substantial fraction of activated, but not resting T cells adhered to astrocytes after 48 h of culture (Figure 2A). FACS analysis after accutase detachment and dissociation of adherent cells showed a significantly increased T cell-to-astrocyte ratio when activated T cells were present in the co-culture (Figure 2B). As previously shown [4], about 40% of *ex vivo* isolated naïve CD4 cells were CD73^+^ and did not express CD39 (Supplementary Figure S1A). However, CD39 was slightly upregulated upon *in vitro* activation with CD3 and CD28 antibodies (Figure 2C, left). Notably, the percentage of positive cells as well as CD39 and CD73 mean fluorescent intensities (MFIs) were significantly and progressively increased in T cells that were shifted to co-culture with astrocytes (Figure 2C, 2D). These results suggest that astrocytes promote ectonucleotidase expression in recently activated CD4 cells.

### Upregulation of CD39 and CD73 in activated CD4 cells adhering to astrocyte is independent of TCR interaction with MHC class II

**Figure 3:**
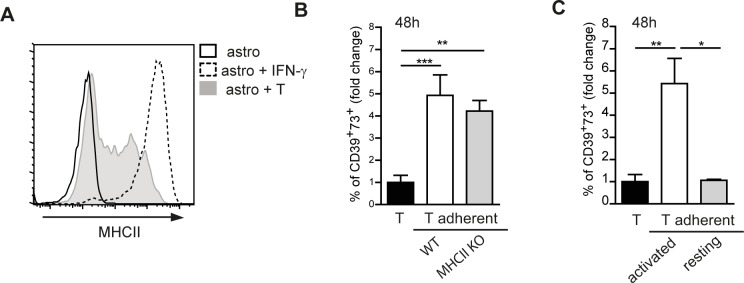
MHC class II independent astrocyte -T cell interaction **A.** MHCII expression on astrocytes alone (black line), stimulated with IFN-γ (dotted line) or co-cultured with T cells (shaded) at 48h of co-culture. **B.** Fold change in percentage of CD39^+^73^+^ T cells adherent to either wild-type (WT) or MHCII deficient astrocytes. Normalized to T cells alone (black bar). One-way ANOVA; Bonferroni's test for comparison among groups; **p < 0.01, ***p < 0.001; *N* = 3. **C.** Fold change in percentage of CD39^+^73^+^ cells in activated or resting T cells adherent to astrocytes after 48h co-culture. Normalized to T cells alone (black bar). One-way ANOVA; Bonferroni's test for comparison among groups; *p < 0.05, **p < 0.01; *N* = 3.

In order to investigate the mechanism through which the contact with astrocytes mediated CD39 and CD73 upregulation in T cells, we initially focused on MHC class II (MHCII) molecules, which are efficiently induced in astrocytes by exposure to IFN-γ [12, 13]. Indeed, we detected MHCII expression in astrocytes co-cultured with activated T cells (Figure 3A), in line with the observation that a substantial fraction of CD4 cells adhering to astrocytes secreted IFN-γ (Supplementary Figure S1B). We addressed whether MHCII in astrocytes was responsible for CD39 and CD73 upregulation in T cells by co-culturing activated CD4 cells with MHCII deficient astrocytes. These cells were equally competent to MHCII sufficient cells in inducing CD39 and CD73 in T cells, thus indicating that the observed phenotype was independent of MHCII mediated interaction (Figure 3B). Analysis of resting CD4 T cells co-cultured with astrocytes revealed lack of difference in the expression of CD39 and CD73 compared to T cells cultured alone (Figure 3C). This observation suggests that only activated T cells efficiently upregulate CD39 and CD73 upon interaction with astrocytes. Only a slight increase in surface CD39 and CD73 was detected in astrocytes in response to activated CD4 cells (Supplementary Figure S1C).

### Adhesion to astrocytes is required for upregulation of CD39 and CD73 in T cells

**Figure 4:**
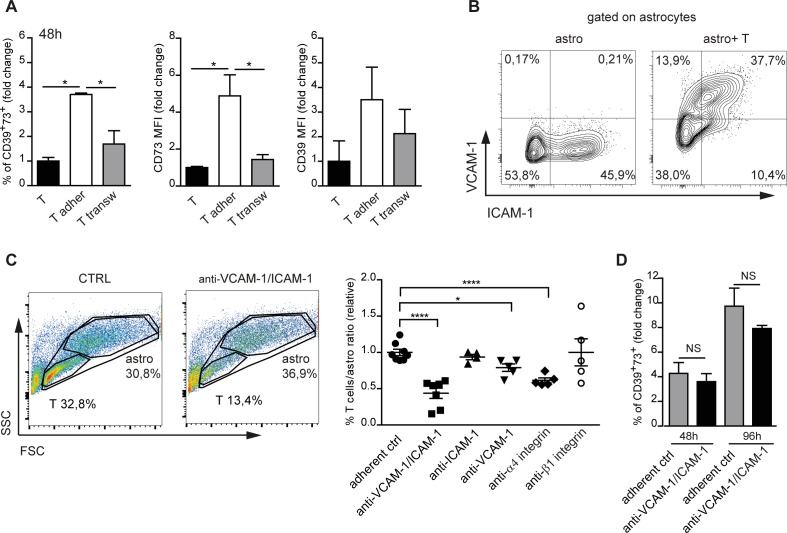
Integrin mediated astrocyte-T cell adhesion **A.** Fold change in percentage of CD39^+^73^+^ T cells either adherent to astrocytes (white bars) or separated by transwell (transw) (gray bars), CD73 and CD39 MFIs after 48h co-culture. Normalized to T cells alone (black bars). One-way ANOVA; Bonferroni's test for comparison among groups; *p < 0.05; *N* = 3. **B.** FACS analysis of VCAM-1 and ICAM-1 expression on electronically gated CD11b^−^CD3^−^ astrocytes either cultured in isolation or co-cultured with T cells for 48h. **C.** FACS analysis of forward scatter (FSC) *vs* side scatter (SSC) of untreated astrocyte-T cell co-culture or co-culture treated with anti-VCAM-1/ICAM-1 antibodies. The indicated regions correspond to T cells and astrocytes with percentages of recovered cells. The panel on the right shows statistics of T cells/astrocytes ratios in the indicated culture conditions. One-way ANOVA; Bonferroni's test for comparison among groups; *p < 0.05, ****p < 0.0001. **D.** Fold change in percentage of CD39^+^73^+^ cells among CD4 cells adherent to astrocytes without (gray bars) or with anti-VICAM-1/ICAM-1 antibodies (black bars) after 48h and 96h culture as compared to T cells cultured in isolation (not shown). One-way ANOVA; Bonferroni's test for comparison among groups; NS = not significant; *N* = 3.

To address whether cell-to-cell contact was required for CD39 and CD73 upregulation in T cells, we co-cultured astrocytes and T cells in a transwell system. Physical separation of the two cell types prevented CD39 and CD73 induction in T cells (Figure 4A). We therefore investigated the possible involvement of T cell/astrocyte adhesion molecules in regulating CD39 and CD73. Upon co-culture with T cells, astrocytes express ICAM-1 (CD54) and VCAM-1 (CD105), which are ligands for LFA-1 (αLβ2) and VLA-4 (α4β1) integrins, respectively [14, 15] (Figure 4B). Pretreatment of T cells with α4 blocking antibodies significantly reduced T cells adhesion to astrocytes, while blockade of β1 integrin was ineffective. On the other hand, anti-VCAM-1 (CD105) antibodies efficiently inhibited T cell adhesion to astrocytes and inhibition was further enhanced by anti-ICAM-1 (CD54) antibodies (Figure 4C). Antibodies addition to cultures did not influence CD4 cell recoveries (data not shown). However, in T cells that still adhered to astrocytes in the presence of blocking antibodies, CD39 and CD73 were efficiently upregulated (Figure 4D). These data suggest that LFA-1 and VLA-4-mediated adhesion as well as downstream signaling were not absolutely required for plasma membrane ectonucleotidases upregulation in T cells, although we cannot exclude that it was impossible to achieve complete inhibition of integrin-mediated adhesion with antibodies.

### Upregulation of CD39 and CD73 in CD4 cells correlates with acquisition of immunosuppressive potential

**Figure 5:**
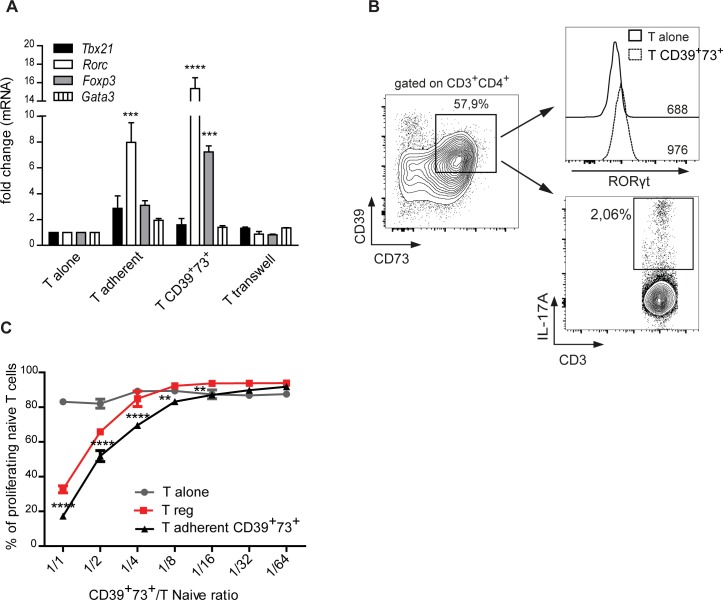
CD39 and CD73 upregulation correlates with immunosuppressive Th17 phenotype **A.** Real-time qRT-PCR for *Tbx21*, *Rorc*, *Foxp*3 and *Gata*3 in CD4^+^ T cells cultured alone, total T cells adherent to astrocytes (T adher), sorted CD39^+^73^+^ T cells adherent to astrocytes or T cells separated by transwell after 48 h co-culture. Two-way ANOVA; Bonferroni's test for comparison with T cells cultured alone, ***p < 0.001, ****p < 0.0001; *N* = 4. **B.** Expression of RORγt (upper histograms with respective MFI indicated) and IL-17A (lower plot) by FACS analysis on CD39^+^73^+^ cells sorted as indicated after 96 h co-culture. **C.** Percentage of UV-labeled proliferating cells in suppression assay with CD4^+^CD39^+^73^+^ T cells detached from astrocytes. Graph shows percentage suppression of proliferation by T cells cultured in isolation (T alone), CD39^+^73^+^ astrocyte-adherent T cells or sorted Treg cells in 72 h culture at different suppressor/responder cells ratio. One representative experiment with triplicates. Two-way ANOVA; Bonferroni's test for comparison among groups, **p < 0.01,****p < 0.0001, *N* = 3.

To functionally characterize CD39 and CD73 expressing T cells generated in co-culture with astrocytes we analyzed the transcription of genes coding for master regulators of CD4 T cell functional polarization. Real-time quantitative reverse transcription PCR (qRT-PCR) performed in the CD39^+^73^+^ subset sorted from cells adhering to astrocytes or on total astrocyte-adherent cells revealed a marked increase in transcription of *Rorc* (encoding RORγt, the master switch transcription factor that defines the Th17 subset) and, to a lesser extent, *Foxp3* (that determines the Treg cells phenotype), whereas transcript levels of *Tbx21* and *Gata-3*, which define Th1 and Th2 T cells respectively, were not significantly different from T cells cultured alone (Figure 5A). We did not detect variations in *Tbx21*, *Rorc*, *Foxp3* or *Gata3* transcripts in CD4 cells separated from astrocytes by transwell chamber with respect to T cells cultured in isolation (Figure 5A). These results indicate that the observed transcriptional regulation required T cell contact with astrocyte. No significant differences in CD4 cell proliferation were observed in the different culture conditions (Supplementary figure S2A, S2B). Flow cytometry confirmed that the majority of CD39^+^73^+^ T cells adhering to astrocytes upregulated RORγt protein after 96h of co-culturing (Figure 5B, upper panel). RORγt is the master transcriptional regulator of Th17 effector functions, however, we detected few cells expressing IL-17A, the cytokine characterizing this subtype (Figure 5B lower panel). The observed phenotype was reminiscent of a recently described immunosuppressive Th17 cell subset [16]. Moreover, qRT-PCR revealed upregulation of both *IFNG* and *IL10* transcripts in CD39^+^73^+^ T cells adhering to astrocytes (Supplementary Figure S2C). We investigated whether CD39^+^73^+^ cells were able to suppress the proliferation of TCR stimulated T cells *in vitro*. We isolated astrocyte-adherent CD4 cells and further sorted CD39^+^73^+^ cells at FACS. Co-culture of these cells with sorted CD4^+^CD44^−^CD62L^+^CD25^−^ naïve T cells stimulated with anti-CD3 antibodies in the presence of irradiated splenocytes from *cd3e^−/−^* mice, resulted in inhibition of T cell proliferation. The efficiency of this inhibition was superior to *Foxp3* expressing Treg cells isolated from *Foxp3*^EGFP^ transgenic mice (Figure 5C). Indeed, only around 50% of *ex vivo* sorted CD25^high^Foxp3^+^ Treg cells expressed CD39 and CD73 (Supplementary Figure S3A), suggesting that enhanced effect of astrocytes adherent cells could be due to selection of ectonuleotidases expressing cells. Collectively, these data indicate that recently activated CD4 T cells acquire a CD39^+^73^+^ Th17 immunosuppressive phenotype upon interaction with astrocytes.

### Role of TGF-β in ectonucleotidase upregulation in T cells

**Figure 6:**
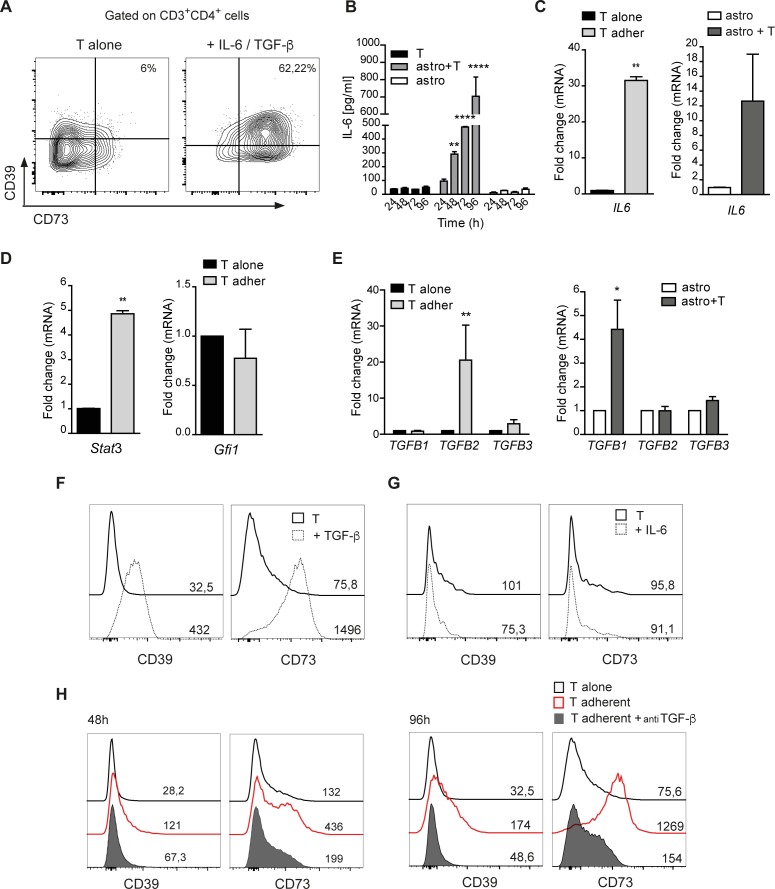
TGF-β promotes CD39 and CD73 upregulation in T cells **A.** FACS analysis for plasma membrane CD39 and CD73 in naive CD4^+^ T cells stimulated for 96 h with anti-CD3/CD28 mabs in the presence of TGF-β and IL-6. **B.** Quantification of IL-6 by ELISA in the supernatant of T cells, astrocytes and astrocyte/T cell co-culture. Two-way ANOVA; Bonferroni's test for comparison among groups, **p < 0.01, ****p < 0.0001; *N* = 3. **C.** *IL6* mRNA levels in purified T cells (left) or astrocytes (right) after 48 h of co-culture. Mann-Whitney's test, **p < 0.01; *N* = 3. **D.** Real-time qRT-PCR for *Stat*3 and *Gfi1* in T cells alone and CD39^+^73^+^ T cells adherent to astrocytes. Mann-Whitney's test; **p < 0.01; *N* = 3. **E.** Expression of *TGFB1*, *TGFB2*, *TGFB3* assessed by qRT-PCR in T cells (left) (*N* = 4) or astrocytes (right) (*N* = 2) alone or co-cultured for 48 h. Mann-Whitney's test; *p < 0.05; ***p < 0.01. **F.**,**G.** FACS analysis of CD39 and CD73 expression with MFIs indicated, in CD3/CD28 stimulated CD4 cells (black line), with addition of TGF-β (F) or IL-6 (G) (dashed line) for 96 h. **H.** CD39 and CD73 expression (MFIs) on CD4^+^ T cells cultured alone (black line) or pre-incubated (filled grey) or not (red) with TGF-β RI kinase inhibitor VI before co-culturing with astrocytes for 48 and 96 h.

As previously described [16], treatment with exogenous IL-6 and TGF-β induced prominent ectonucleotidase upregulation in CD4 cells, mimicking the induction triggered by astrocytes (Figure 6A). Analysis of IL-6 in the culture supernatant showed the progressive increase of the cytokine concentration in T cell/astrocyte co-cultures over time, whereas the cytokine was barely detectable in cultures of T cells and astrocytes grown in isolation (Figure 6B). Analysis of *IL6* transcripts showed that mRNA levels were increased in both T cells and astrocytes in co-culture (Figure 6C). Since immunosuppressive Th17 cells are characterized by transcriptional upregulation of *Stat3* and downregulation of the transcription factor *Gfi1*, we analyzed by real-time qRT-PCR *Stat3* and *Gfi1* genes. Figure 6D shows that, analogously to the cell subset described by Chalmin et al. [16], CD4 cells adhering to astrocytes significantly upregulated *Stat3* and downregulated *Gfi1*, albeit non-significantly. To explore the possible involvement of TGF-β in T cell polarization evoked by interaction with astrocytes, we first analyzed *TGFB* transcript levels by real-time qRT-PCR in astrocytes and T cells either co-cultured or cultured in isolation. Cell co-culturing resulted in higher levels of *TGFB1* and *TGFB2* transcripts in astrocytes and T cells, respectively (Figure 6E). Interestingly, incubation of recently activated CD4 cells with TGF-β alone was sufficient to promote CD39 and CD73 upregulation (Figure 6F), whereas addition of IL-6 to cultures did not result in CD39 and CD73 variations (Figure 6G). Consistently, addition of TGF-β RI kinase inhibitor VI significantly inhibited both CD39 and CD73 expression in adherent CD4 T cells at 48 (left) and 96h (right) (Figure 6H). Altogether, these data point to TGF-β as the key secreted molecule responsible for astrocyte-dependent CD39 and CD73 upregulation in the T cell.

### Modulation of spontaneous calcium oscillations in astrocytes by CD39 expressed in T cells

**Figure 7:**
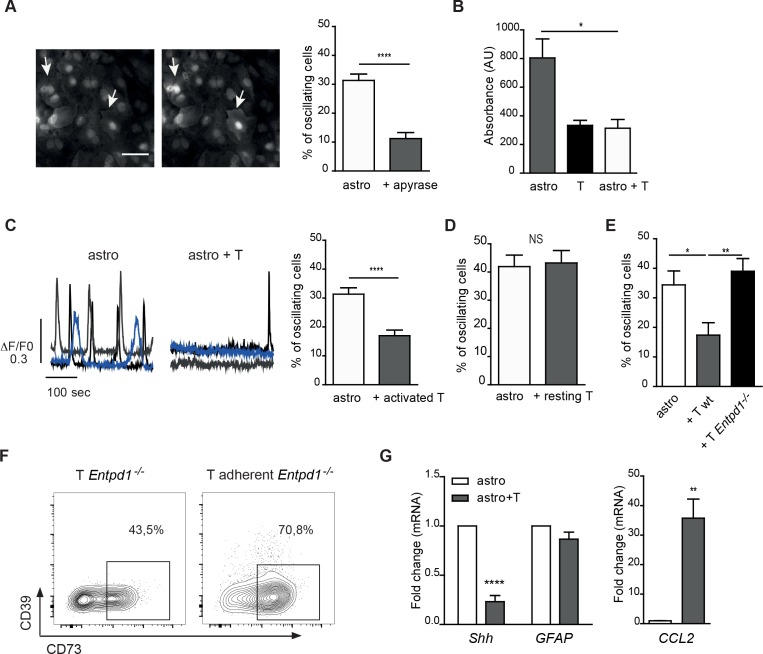
CD39 mediated inhibition of spontaneous cytosolic Ca^**2+**^ oscillations in astrocytes by recently activated CD4 cells **A.** Representative fluorescence images of astrocytes loaded with Oregon green showing spontaneous calcium oscillations (left, arrows) and statistical analysis of astrocytes showing calcium oscillations in control culture (astro) or with addition of apyrase. Mann-Whitney's test; ****p < 0.0001; *N* = 3. **B.** Luciferin/luciferase assay for extracellular ATP concentration in medium from astrocyte or T cell cultures, or astrocyte/T cell co-cultures (after 72 h). Means of triplicates ± SEM from a representative experiment are shown. One-way ANOVA; Bonferroni's test for comparison among astrocytes cultured alone and astrocytes in co-culture with T cells; *p < 0.05. **C.** Temporal analysis of cytosolic Ca^2+^ concentration recorded from three representative astrocytes cultured alone (left) or co-cultured with activated T cells for 96 h (right) and statistical analysis of astrocytes showing spontaneous calcium oscillations. Mann-Whitney's test; ****p < 0.0001, *N* = 6. **D.** Same analysis as in C but with resting T cells. Student's t-test, NS = not significant; *N* = 2. **E.** Same analysis as in C with activated T cells from wild-type and *Entpd1*^−/−^ mice. One-way ANOVA; Bonferroni's test for comparison among groups; *p < 0.05; **p < 0.01; *N* = 3. **F.** FACS analysis of CD39 and CD73 expression in activated *Entpd1*^−/−^ T cells either cultured alone or adhering to astrocytes after 48 h co-culture. **G.** Real-time qRT-PCR for *CCL2*, *GFAP* and *Shh* in astrocytes either cultured alone or co-cultured with activated T cells (at 48 h). Mann-Whitney's test, **p < 0.01, ****p < 0.0001; *N* = 4.

It is known that astrocytes communicate among themselves and with other brain cells through regulated increases in intracellular calcium concentrations [17-20], which are sustained by extracellular ATP [reviewed in [21]]. Indeed, addition of the ATP hydrolyzing enzyme apyrase to astrocyte cultures resulted in significant reduction of the percentage of cells showing Ca^2+^ oscillations (Figure 7A, right). Hence, we reasoned that ATP hydrolysis by CD39^+^73^+^ T cells might negatively modulate Ca^2+^ signaling in astrocytes. Accordingly, significantly lower extracellular ATP levels were detected in astrocytes co-cultured with T cells compared to astrocytes cultured alone (Figure 7B). A two-fold decrease in the percentage of astrocytes showing spontaneous calcium oscillations was detected in astrocytes co-cultured with CD39^+^73^+^ T cells (Figure 7C and Supplementary Movies S1-2) but not resting CD4 cells (Figure 7D). Furthermore, co-culture of activated *Entpd1*^−/−^ T cells with astrocytes resulted in unaltered percentage of astrocytes displaying Ca^2+^ oscillations, indicating that CD39 in T cells adhering to astrocytes inhibits purinergic Ca^2+^ signaling in astrocytes (Figure 7E). The lack of effect of *Entpd1*^−/−^ T cells on astrocyte calcium dynamics was not due to impaired capacity of T cells to interact with astrocytes, as the number of *Entpd1*^−/−^ T cells adhering to astrocytes was comparable to wild-type T cells (Supplementary Figure S3B). Finally, we observed upregulation of plasma membrane CD73 in *Entpd1*^−/−^ CD4 cells adhering to astrocytes, indicating that CD73 is regulated independently of CD39 expression (Figure 7F). These results indicate that the reciprocal communication between recently activated CD4 cells and astrocytes results in a CD39-dependent reduction of ATP-mediated Ca^2+^ activity in astrocytes. Consistent with a functional consequence of this signaling circuit in astrocytes, we correlated the reduction of spontaneous Ca^2+^ oscillations in astrocytes with a decrease in *sonic hedgehog* (*Shh*) expression, a secreted molecule that promotes BBB integrity [22] and to enhanced transcription of the gene encoding the chemokine CCL2, which triggers immune cell infiltration [23, 24] (Figure 7G). Therefore, astrocyte interaction with activated CD4 cells would implement a transcriptional program promoting BBB permeability and leukocyte infiltration.

## DISCUSSION

The CNS has been considered for long time as an immune privileged site. However, the detection of activated T cells in the brain parenchyma in healthy conditions indicated that these cells could in fact cross the BBB. Within this context, astrocytes have been generally considered to play a beneficial role as immunosuppressive and neuroprotective elements. Nevertheless, astrocytes were shown to be capable of producing a range of proinflammatory cytokines and acting as antigen presenting cells (reviewed in ref [25]). A number of efforts have been devoted at defining functional polarization of CD4 cells upon interaction with astrocytes with conflicting results [26-30]. Treg cells were shown to develop upon contact with astrocytes and administration of these cells alleviated CNS inflammation and clinical symptoms in EAE [31]. Yet, the molecular features of the immunosuppressive arm eventually triggered by astrocytes are so far unknown. In this study we aimed at addressing the outcome of the interaction between CD4 cells and astrocytes in a possible physiological as well as pathophysiological context. To this end we analyzed *in vitro* molecular and functional clues resulting from the interaction of recently activated CD4 cells with astrocytes. This interaction resulted in upregulation of VCAM-1 and ICAM-1 in astrocytes, which promoted ectonucleotidase expression in T cells. The humanized anti-α4 integrin antibody natalizumab has shown therapeutic efficacy in relapsing-remitting MS by affecting T cells infiltration of the brain [32]. Interestingly, blockade of α4 integrin also inhibited T cells adhesion to astrocytes in our assay, suggesting that natalizumab might limit astrocyte/T cell interaction. We defined the Th17-like immunosuppressive phenotype resulting from this interaction that might constitute a feed-back mechanism for limiting tissue damage by brain infiltrating pro-inflammatory T cells. Notably, a population of IL-4 producing CD4 cells was recently shown to promote neuroprotection after CNS injury in an antigen independent fashion [33]. This study together with ours suggest that therapeutic strategies aimed at converting brain infiltrating T cells to a neuroprotective function can be envisaged.

We have shown that TGF-β is crucially involved in astrocyte-dependent CD39 and CD73 upregulation in the T cell. These ectonucleotidases contribute to CD4 cell immunosuppressive function [5-7]. Upregulation of TGF-β1 in astrocytes characterizes the early phases of MS and EAE, and promotes brain infiltration by myelin oligodendrocyte glycoprotein- (MOG-) specific pro-inflammatory CD4 cells [34]. Interestingly, administration of complete Freund adjuvant (CFA), a potent inducer of TGF-β1 in astrocytes [34] before induction of EAE protects from the disease [35]. These observations suggest that TGF-β1 produced by astrocytes might also exert a protective role in EAE. The functional conversion of activated T cells by TGF-β might represent an immunoregulatory mechanism to control tissue inflammation in the absence of acute antigen recognition.

Different signaling mechanisms can skew astrocyte function at the BBB resulting in opposite outcomes, such as promoting or limiting neuroinflammation [2]. Hydrolysis of extracellular ATP by T cell ectonucleotidases may have important implications for microvessel permeability, independently of adenosine generation, by affecting astrocyte Ca^2+^ activity. Indeed, Ca^2+^ signaling in astrocyte foot processes can influence blood flow through activation of phospholipase A2 (PLA2), arachidonic acid (AA) mobilization and production of vasoactive AA metabolites [36-40], with Ca^2+^ elevations being more common in vasoconstriction [41]. Interestingly, the reduction of spontaneous Ca^2+^ oscillations in astrocytes was associated to decrease in *Shh* expression, a secreted molecule that promotes BBB integrity and to enhanced transcription of *CCL2*, which triggers immune cell infiltration. These observations suggest that MHC-independent bidirectional signaling between astrocytes and T cells may favor T cell polarization toward an immunosuppressive phenotype as well as promote T cells infiltration into the brain tissue. This regulatory path might be important to resolve inflammation determined by infectious processes upon local clearance and/or limited presentation of pathogenic antigen/s.

In relapsing-remitting MS, episodes of acute inflammation, tissue damage and neurological dysfunction are followed by deactivation, repair and clinical remission [42]. In contrast, the progressive form of the disease is characterized by axonal degeneration and irreversible disability that are relatively independent from inflammation [43]. The oscillatory nature of relapsing-remitting MS is the result of the local homeostatic regulation of T cell activation. The signaling pathways leading to this remarkable capacity of suppressing inflammation and promoting repair mechanisms in the brain could be exploited therapeutically to limit the immunopathological damage that can ultimately lead to the progressive form of the disease [44]. We hypothesize astrocytes-mediated upregulation of CD39 and CD73 in activated CD4 cells is part of this adaptive regulatory framework at the BBB. The Th17-like immunosuppressive activity induced by contact with astrocytes might contribute a beneficial immunomodulatory effect during neuroinflammation as well as oppose to resolution of chronic infections and anti-tumor T cell responses.

## MATERIALS AND METHODS

### Animals and ethics statement

Mice were bred at Humanitas Research Institute in Milan, Italy and Institute for Research in Biomedicine in Bellinzona, Switzerland. C57BL/6J (H-2^b^) and congenic *FoxP3*^EGFP^ (B6.Cg-Foxp3tm2Tch/J) and *Cd3e^−/−^* mice were from Jackson Lab, *Entpd1^−/−^* mice were kindly provided by Dr. Simon C. Robson (Beth Israel Deaconess Medical Center, Boston, MA). Animals were kept under EU directives (2010/63/EU) and the Italian Legislation (L.D. 26/2014) for animal research with protocols approved by Institute Ethical Committee and the Italian Ministry of Health (162/2011-B). In Switzerland, all animal experiments were performed in accordance with the Swiss Federal Veterinary Office guidelines and authorized by the Animal Studies Committee of Cantonal Veterinary.

### Cells

CD4^+^ cells were isolated from the spleen and lymph nodes of 2-4 months old female mice using magnetic beads coupled to anti-CD4 antibodies (Miltenyi Biotec) and sorted by FACS (FACSAria, Beckton Dickinson) as CD4^+^25^−^62L^+^44^−^ naive T cells. Alternatively, *Foxp*3^+^ Treg cells were isolated from *Foxp3^E^*^GFP^ mice by sorting for CD25 and EGFP. Purified CD4^+^ cells were stimulated for 40 h with plate-bound anti-CD3 (10 μg/ml) and anti-CD28 (5 μg/ml) antibodies (Biolegend) prior to transfer onto purified primary astrocyte cell cultures. Astrocytes were obtained from neonatal pups (postnatal day 1-3) and pure cultures of astrocytes (> 99.5%) were obtained by shaking flasks for 24 h at 37°C at days 2 and 6 after plating. Resting or polyclonally activated T cells were plated on astrocyte monolayer, at an astrocyte:T cell ratio of 1:0.5 in the presence of murine IL-2 (90 U/ml). Co-cultures were maintained in RPMI-1640 medium supplemented with L-glutamine, non-essential amino acids, sodium pyruvate and 5% FBS. In a set of experiments astrocytes were treated with IFN-γ (25 ng/ml, Peprotech) for 16 h. CD4 T cells were differentiated *in vitro* by IL-6 and TGF-β (R&D) as previously described (16). In distinct set of experiments, CD4^+^ cells were plated in transwell culture inserts (0.4 μm pore, Costar) or astrocytes were pre-treated with anti-ICAM-1 (10 μg/ml) and VCAM-1 (10 μg/ml) antibodies for 4 h (Biolegend) to prevent T cells adhesion. Alternatively, CD4 cells were incubated with α4 integrin blocking antibody (CD49d, R1-2) (50 μg/ml) or with β1 integrin blocking antibody (CD29, HMβ1-1) (25 μg/ml). To block TGF-β receptor, activated CD4 cells were pre-incubated for 3 h with 10 μM TGF-β RI kinase inhibitor VI (SB 431542 hydrate, Sigma Aldrich).

### Antibodies, flow cytometry and ELISA

After 48 h or 96 h of incubation, non-adherent CD4 T cells were removed by washing the co-culture 3 times with PBS (2% FBS). The remaining adherent cells (T cells and astrocytes) were incubated with accutase (Millipore) at 37°C for 40 min, collected and analyzed by flow cytometry. CD4^+^ cells were stained with the following mouse specific monoclonal antibodies: CD3 (clone 17A2), CD4 (GK1.5), MHCII (M5/114.15.2), CD39 (24DMS1), Rorg (gamma, greek letter) t (lower case) (AFKJS-9), IFN-γ (XMG1.2), CD25 (PC61 5), FoxP3 (FJK-165), CD44 (IM7), CD62L (MEL-14) (eBioscience), CD73 (TY11.8) and IL-17A (TC11-18H10.1) (Biolegend). Astrocytes were stained for CD11b (M1/70) (Biolegend), VCAM-1 (429) (BD Bioscience), ICAM-1 (YN1/1.7.4) (eBioscience). All samples were acquired with a FACSCalibur or FACSCanto (Becton Dickinson) and analyzed with FlowJo software (FlowJo v10.0.7). IL-6 protein concentration was determined using the DuoSet ELISA Development System according to the manufacturer's instructions (R&D Systems).

### Immunosuppression assay

Immunosuppression assay was performed as previously described [45]. Briefly 50,000 naïve CD4 T cells were labeled with Ultraviolet (UV) (Molecular Probes) and stimulated with CD3 monoclonal antibody (0.5 μg/ml) in the presence of 125,000 irradiated splenocytes from *cd3e^−/−^* mice. Serial dilutions of Treg cells or purified CD39^+^73^+^ cells adhering to astrocytes were added to cultures. UV dilution in naïve T cells was analyzed at 72 h. Acquisitions at FACS were standardized by fixed numbers of calibration beads (BD Pharmingen).

### Real-time PCR

Quantitative real-time PCR (qRT-PCR) was performed as previously described [46]. RNA was isolated from cell cultures using the PureZOL RNA Isolation Reagent (BioRad), reverse transcribed to cDNA and qRT-PCR was performed using the Applied Biosystems ViiA™ 7 RT-PCR System. Experimental cDNA was amplified by qRT-PCR where a target cDNA (e.g., *Rorc, FoxP3, TGFB1*) and a reference cDNA (*18S*, *GAPDH* and *HPRT*) were amplified simultaneously using an oligonucleotide probe with a 5′ fluorescent reporter dye (6-FAM). Data were analyzed using the comparative threshold cycle (Ct) method and results are expressed as fold difference.

### Calcium imaging

Calcium imaging was performed as previously described on cells loaded with Oregon Green (Molecular Probes) [47]. Briefly, astrocytes were loaded with the calcium sensitive dye Oregon Green for 1 h at 37°C in medium and then imaged for calcium activity. The recording was performed in KRH (Krebs'-Ringer's-HEPES) containing (in μM): 125 NaCl; 5 KCl; 1.2 MgSO_4_; 1.2 KH_2_PO_4_; 25 HEPES; 6 Glucose; 2 CaCl_2_; pH 7,4. Recording chambers were placed on the stage of an IX-71 inverted microscope (Olympus) equipped with an EMCCD (electron-multiplying CCD) camera (Quantem 512×512, Photometrics). Illumination was obtained using a light-emitting diode single LED (Cairn Research Optoled; 470nm) and a related GFP filter, 16 bit images were captured using a 40X objective [numerical aperture (NA): 1.3]. Regions of interest (ROIs) of about 15-pixel diameter (corresponding to about 12 μm) were drawn on the cell cytoplasm of virtually all the cells in the recorded field. Time-lapse recording of calcium dynamics was obtained with an acquisition rate of 2 Hz for 500 seconds, and on-line acquisition and off-line analysis were performed with MetaFluor software (Molecular Devices). Oscillating astrocytes displayed peak ΔF larger than 10 standard deviations of mean baseline noise. The average of F0 was comparable among astrocytes under different conditions. Roughly 20-25 astrocytes were analyzed in each recording field.

### ATP measurment

Cell culture supernatants were measured using a luciferin/luciferase assay (Molecular Probes, Leiden, NL) according to the manufacturer's instruction [48]. To degrade extracellular ATP in astrocytes cultures, cells were incubated for 1 h with the ATP hydrolyzing enzyme Apyrase (30U/ml, Sigma Aldrich).

### Non-relapsing rodent EAE

Non-relapsing EAE (nr-EAE) was induced in female C57Bl/6 mice by immunization with 200 mg/mouse of MOG 35-55 (Espikem, Florence, Italy) and 2 injections of pertussis toxin (500 ng/mouse) the day of immunization and 48 h later. Clinical score was assigned according to a standard 0 to 5 scale, as described [49]. For analysis of brain infiltrating T cells at FACS, mice were perfused with HBSS, brains were chopped and digested in Collagenase D/DNase I (Roche) for one hour at 37°C with shaking. Tissues were resuspended in 35% Percoll and then centrifuged 40 min at 400 g to obtain the sediment fraction.

### Immunostaining and confocal microscopy

Fixed 10 μm-thick frozen brain and spinal cord section were immunostained and acquired with an Olympus Fluoview FV1000 laser scanning confocal microscope in a channel mode with 405, 488, 568, and 647 nm excitations. Sections were imaged with oil immersion lens 40× (NA 1.3) in xy optical sections (1024×1024 pixels) of 0.3 μm. The z-stacks were reconstructed and analyzed by ImageJ Software (1.48v, Java).

### Statistical analysis

Values, expressed as mean ± SEM, were obtained from at least 3 independent experiments. Differences between multiple groups were analyzed by one-way or two-way ANOVA with a Bonferroni's post hoc test. Comparisons between two groups following a normal distribution were analyzed using an unpaired *t*-test (two-tail distribution) or a Mann-Whitney test as indicated in each figure. Statistical analysis was performed using GraphPad Prism (Graph-Pad Software).

## SUPPLEMENTARY MATERIAL FIGURES AND VIDEOS






