# Supplementary material for: Ectonucleotidase activity and immunosuppression in astrocyte-CD4 T cell bidirectional signaling
Source: Oncotarget. 2016 Jan 13;7(5):5143–56. doi: 10.18632/oncotarget.6914 (PMC4868677; doi:10.18632/oncotarget.6914)
Supplement: Supplementary file 1 [file oncotarget-07-5143-s001.pdf]

Ectonucleotidase activity and immunosuppression in astrocyte-CD4 T cell bidirectional signaling

Supplementary Material

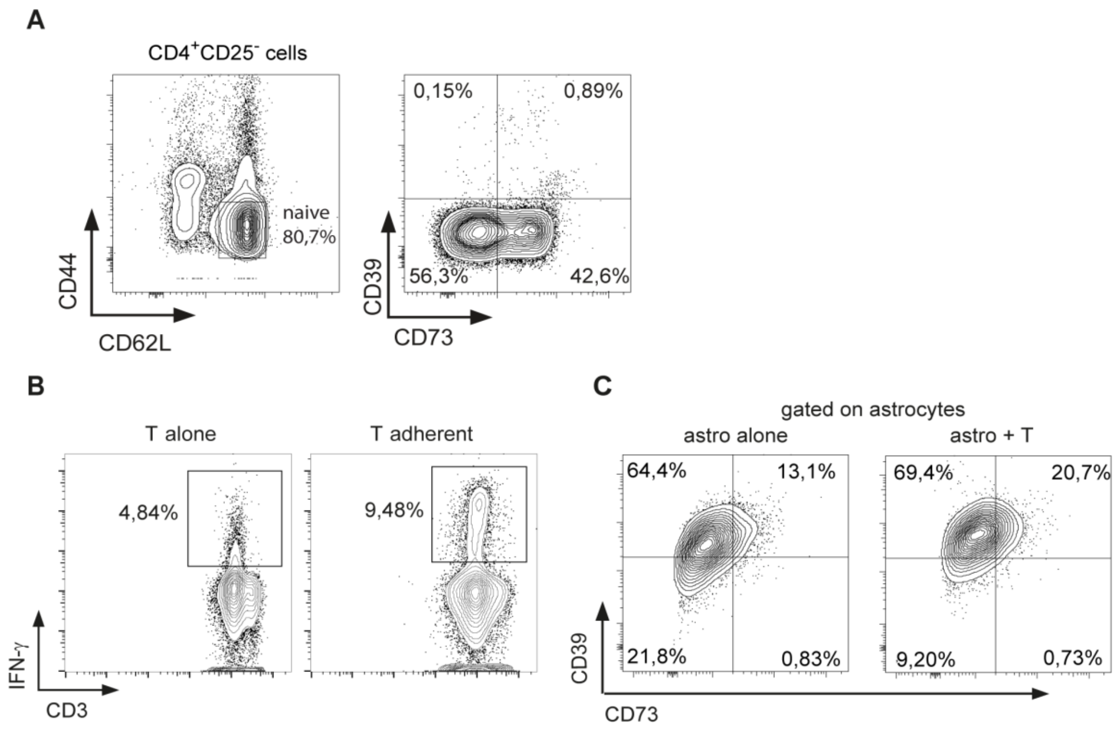

Supplementary Figure 1

S1A Representative counter plot of *ex vivo* isolated CD4 positive T cells. Naïve cells population gated as $CD62L^{high}CD44^{low}$ was analyzed for CD39 and CD73 expression.

S1B Representative intracellular staining for IFN-γ in T cells cultured in isolation or after 96 h of co-culture with astrocytes.

S1C Representative counter plot showing surface staining of CD39 and CD73 in $CD11b^{-}CD3^{-}$ astrocytes either in isolation or co-cultured with T cells for 48 h.

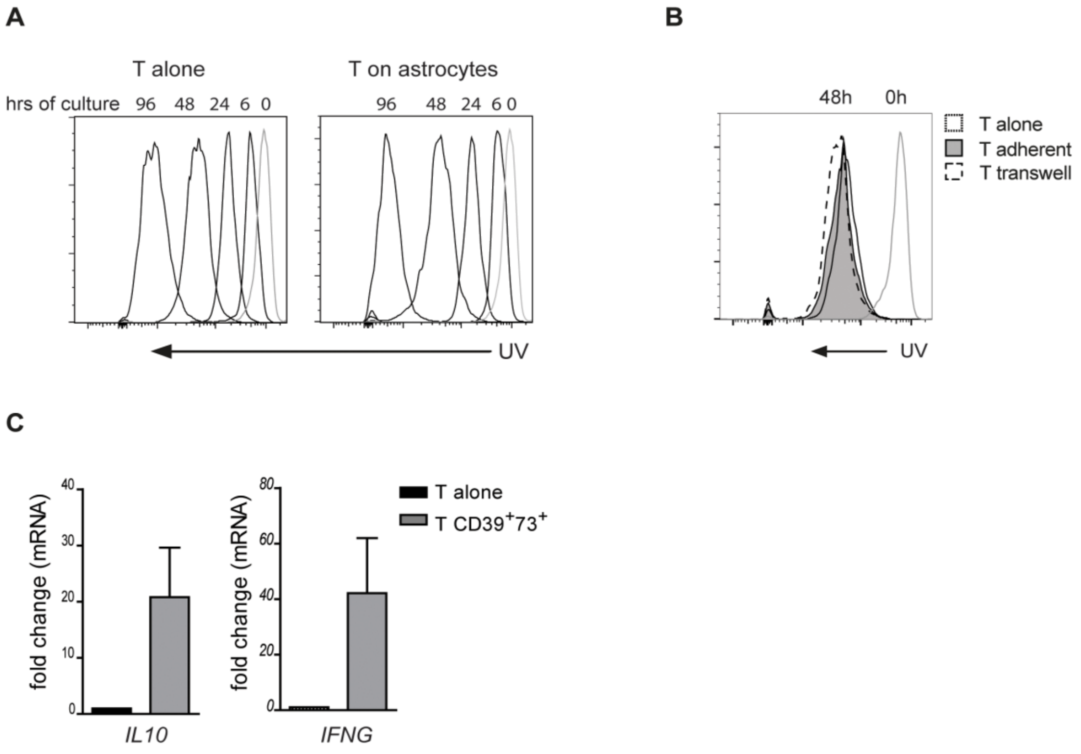

Supplementary Figure 2

S2A Activated T cells labeled with Ultraviolet were cultured as indicated and analyzed at 6, 24, 48 and 96 hours.

S2B Analysis of Ultraviolet dilution in T cells either cultured in isolations, adhering to astrocytes or in transwell at 48 h of culture.

S2C Real-time qRT-PCR for *IL10* and *IFNG* in T cells cultured in isolation or CD39^{+}73^{+} T cells adherent to astrocytes.

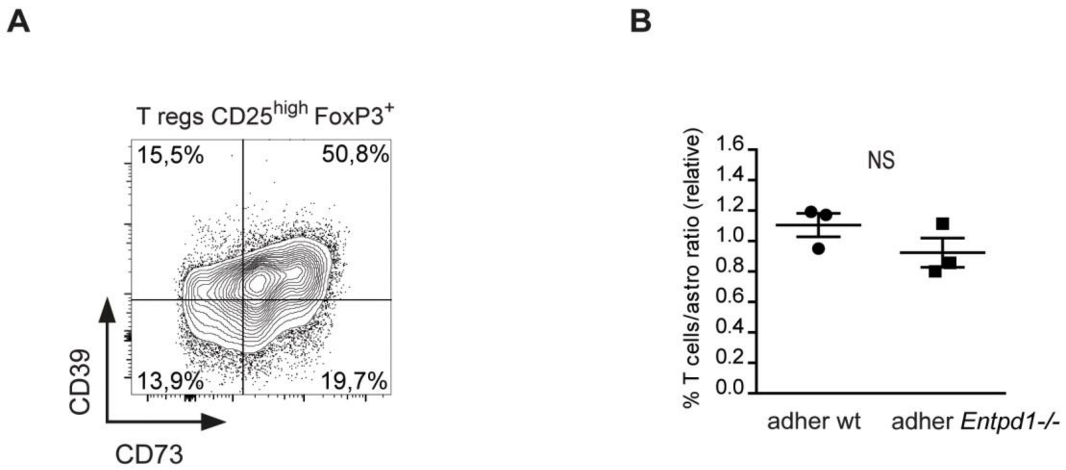

Supplementary Figure 3

S3A Representative counter plot showing surface staining of CD39 and CD73 in *ex vivo* sorted Treg cells

S3B Statistical analysis of wild-type vs *Entpd1*$^{-/-}$ T cells to astrocytes ratio after accutase treatment at 48 h of co-culture. NS: not significant
